# Relationship between gut hormones and glucose homeostasis after bariatric surgery

**DOI:** 10.1186/1758-5996-6-87

**Published:** 2014-08-16

**Authors:** Priscila Campos Sala, Raquel Susana Torrinhas, Daniel Giannella-Neto, Dan Linetzky Waitzberg

**Affiliations:** Medical School, Department of Gastroenterology, Digestive Surgery Discipline (LIM 35), University of São Paulo, Av. Dr. Arnaldo, 455, Cerqueira César, CEP: 01246-903, São Paulo, Brazil; University Nove de Julho, São Paulo, Brazil

**Keywords:** Type 2 diabetes mellitus, Obesity, Bariatric surgery, Gut hormones, Enteroendocrine cells, Glucose homeostasis

## Abstract

Type 2 diabetes mellitus (T2D) is emerging as a worldwide public health problem, and is mainly associated with an increased incidence of obesity. Bariatric surgery is currently considered the most effective treatment for severely obese patients. After bariatric surgery, T2D patients have shown a significant improvement in glycemic control, even before substantial weight loss and often discontinuation of medication for diabetes control. A central role for enteroendocrine cells from the epithelium of the gastrointestinal tract has been speculated in this postoperative phenomenon. These cells produce and secrete polypeptides - gut hormones - that are associated with regulating energy intake and glucose homeostasis through modulation of peripheral target organs, including the endocrine pancreas. This article reviews and discusses the biological actions of the gut hormones ghrelin, cholecystokinin, incretins, enteroglucagon, and Peptide YY, all of which were recently identified as potential candidates for mediators of glycemic control after bariatric surgery. In conclusion, current data reinforce the hypothesis that T2D reversion after bariatric surgery may be related to glycemic homeostasis developed by the intestine.

## Introduction

Type 2 diabetes mellitus (T2D) and obesity are major and increasingly common global health problems [1–4] that are often associated with each other. This relationship involves two main defects: insulin resistance (i.e., decreased capacity of insulin to stimulate glucose uptake in insulin-dependent tissues and to suppress endogenous glucose release, mainly from the liver) and β cell dysfunction resulting in decreased insulin secretion [5]. The systemic insulin resistance in obesity can be initiated largely in adipose tissue. Macrophage-mediated tissue inflammation is a core mechanism of dysfunction in adipose tissue. Adipose tissue can communicate with the liver and other organs, such as, muscle and pancreas, and release proinflammatory cytokines leading to reduction of insulin sensitivity [6].

Intervention programs to promote a healthy lifestyle, such as nutritional therapy, physical exercises, and pharmacotherapy, are extensively used in several combinations to fight obesity. Unfortunately, with very rare exceptions, the weight loss is usually quite modest, especially in severely obese persons [5, 7, 8]. Bariatric surgery is increasingly being acknowledged as an efficient method of treating severely obese patients (Body Mass Index [BMI] ≥40 kg/m^2^) and/or patients with a grade II obesity (BMI ≥35) to achieve the remission of obesity-associated comorbidities, including T2D [9–11].

There are many different surgical procedures aiming body weight loss in severely obese patients, and they can be classified as techniques restrictives (e.g., reduction of gastric volume, such as AGB and SG), malabsorptives (e.g., intestinal bypass, such as DJB), or mixed (combination of restrictive and malabsorptive techniques, such as BPD, BPD-DS and RYGB), as follows in Table 1.

**Table 1:** **Bariatric surgeries: mainly types and descriptions**

Principle	Type	Description
Restrictive	Adjustable gastric band (AGB)	Involves an inflatable band that is placed around the upper portion of the stomach, creating a small stomach pouch above the band, and the rest of the stomach below the band. The size of the stomach opening can be adjusted by filling the inflatable band with sterile saline, which is injected through a port placed under the skin [12].
	Sleeve gastrectomy (SG)	Also known as vertical gastrectomy. The procedure removes the gastric fundus and body, leaving a gastric tube along the lesser curve [13].
Malabsorptive	Duodenojejunal bypass (DJB)	The pylorus is preserved and the length of the biliopancreatic limb is 70 cm from the ligament of Treitz. The Roux limb is 100 cm long. The duodenum and jejunum are bypassed for the nutrient flow. This technique is presently an experimental procedure [9, 14, 15].
Mixed	Biliopancreatic diversion (BPD)	This technique consist in a reduction of gastric pouch (70±10 mL), an alimentary limb composed of 400 cm, a common limb of 100 cm, and a biliopancreatic limb, the remainder of the small intestine [16].
	Biliopancreatic diversion with duodenal switch (BPD-DS)	Adaptation from the biliopancreatic diversion. is a procedure with two components. First, a smaller, tubular stomach pouch is created by removing 70% of portion of the stomach, very similar to the sleeve gastrectomy. Next, a large portion of the small intestine is bypassed [12, 17–19].
	Roux-en-Y gastric bypass (RYGB)	Consists in the reduction of the gastric food reservoir (to a capacity of 30 to 50 mL) and excludes the passage of nutrients through the remaining stomach, duodenum, and proximal jejunum, with an isolated Y-shaped jejunal loop being anastomosed to the small stomach pouch [20, 21].

Notably, glycemic homeostasis after bariatric surgery depends on the kind of surgical technique applied. After restrictive procedures, glycemic homeostasis takes longer to establish and is largely related to weight loss [15], while full T2D remission is observed within days or a few weeks [10, 22, 23] after malabsorptive or mixed surgical procedures [24], even before significant weight loss takes place. Currently, Roux-en-Y gastric bypass (RYGB) is considered the gold standard technique among the bariatric surgery procedures [11, 20, 21].

The T2D remission rate with the use of a restrictive surgical technique, such as, AGB is 48%, compared to 84% after RYGB. The hypoglycemic effects of malabsorptive or mixed surgical procedures appear not to depend only on body weight loss [25–28].

The gastrointestinal tract is quite important for the control of energetic homeostasis. Enteroendocrine cells from the coating epithelium of the gastrointestinal tract produce and secrete polypeptides that can activate neural circuits that, in turn, communicate to peripheral organs, including the liver, muscular tissue, adipose tissue, and islets of Langerhans in the pancreas. Through such properties, these hormones may play important roles in food control and in glycemic homeostasis [6, 14, 29–32].

To explain the early remission of T2D after bariatric surgery, we recently reviewed hypotheses proposing that anatomical changes arise from malabsorptive procedures with metabolic modifications of the small intestine [29]. Here, we discuss evidence linking these metabolic modifications to the biological actions of gut hormones, such as ghrelin, cholecystokinin, incretins, enteroglucagon, and Peptide YY, all of which were recently identified as potential candidates for inducing glycemic control after bariatric surgery, reinforcing the concept of “metabolic surgery” [30].

There are two mainly hypotheses about the mechanisms of T2D remission after bariatric surgery. Both of them involve change in gut hormones: Hindgut hypothesis and foregut hypothesis [29].

The hindgut hypothesis proposed by Cummings et al. [31] suggested that the production of the insulinotropic gut hormones, such as GLP-1 and PYY, are stimulated when nutrients arrive directly to the distal intestine, contributing to the reversion of hyperglycemia, even in the absence of any gastric restriction.

On the other hand, in foregut hypotheses proposed by Rubino, et al. [33], suggested that in susceptible individuals, the surgical deviation of the proximal intestine inhibits the release of diabetogenic signals, such as GIP, which release can be induced by the presence of food in the duodenum. When food stops flowing through the duodenum and the proximal jejunum after RYGB, this/these factor (s), called “anti-incretin,” is/are inhibited. Therefore, the glycemic control contributes to T2D remisson. GIP appears to have a dual function concerning glycemic control; it is able to reduce glycemia (insulinotropic effect), but is also able to increase glycemia (glucagonotropic effect). In T2D patients, GIP has less insulinotropic power, behaving mainly as a glucagonotropic hormone in these patients [34, 35]. Therefore, this hypothesis has been considered of major scientific importance, but its validity still needs to be confirmed. See in Table 2 some mainly studies that correlate changes in intestinal hormones after different surgical techniques.

**Table 2:** Summary of the main changes in gut hormones after bariatric surgery* [13] [16] [36]**-** [40]

	Fasting Ghrelin	Ghrelin (PP)	Fasting CCK	CCK (PP)	Fasting GLP-1	GLP-1 (PP)	Fasting GIP	GIP (PP)	Fasting OXM	OXM (PP)	Fasting PYY	PYY (PP)
AGB	↔ ↑	↔	Ø	Ø	↔	↔	↔	↔	Ø	Ø	↔	↔
SG	↓	↓	↔	↑	↔	↑	Ø	Ø	Ø	Ø	↔ ↑ ↓	↑
BPD	↔ ↑	↔	Ø	Ø	↔ ↑	↑	↓	↓	Ø	Ø	↑	↑ Ø
BPD-DS	↓	Ø	Ø	Ø	Ø	Ø	Ø	Ø	Ø	Ø	↑	↑
RYGB	↔ ↑ ↓	↔↓	↔	↑	↔	↑	↔	↔	↔	↑	↔	↑

Legend - *Evidence was obtained from both human and animal published studies. No studies were found about gut hormones and DJB. *Abbreviations* - *AGB* Adjustable Gastric Band, *VBG* Vertical Banded Gastroplasty, *SG* Sleeve Gastrectomy, *JB* Jejunoileal Bypass, *DJB* Duodenojejunal Bypass, *BPD* Biliopancreatic Diversion, *BPD-DS* Biliopancreatic Diversion with Duodenal Switch, *RYGB* Roux-en-Y Gastric Bypass. ↔: No significant change in the majority of studies; ↑: Significant increased in the majority of studies; ↓: Significant decreased in the majority of the studies; Ø: No studies for this parameter; PP: postprandial; CCK: Cholecystokinin; GLP-1: Glucagon Like Peptide-1; GIP: Glucose-dependent insulinotropic polypeptide; OXM: Oxyntomodulin; PYY: Peptide YY.

Adapted from: [36].

### Ghrelin

Ghrelin is currently the only known intestinal hormone with orexigenic functions [41]. This 28-amino acid protein is derived from pre-proghrelin. Ghrelin is synthesized mainly by the gastric X/A cells and the small intestine—to a lesser extent—based on increased distance from the pylorus. Ghrelin experiences unique post-translational acylation in which serine residue 3 is covalently connected to octanoic acid to form acyl-ghrelin. This acylation is necessary for ghrelin to connect to the growth hormone secretagogue-receptor (GHS-R) and to cross the blood-brain barrier [42]. In the hypothalamus, ghrelin concentrations rise during fasting and before meals to stimulate appetite and digestive secretions [9, 41, 43, 44]. Experimentally, the chronic administration of ghrelin causes hyperphagia and increases adiposity [9].

Glucose homeostasis is also influenced by ghrelin. Ghrelin likely increases plasma glucose through the stimulation of the insulin counter-regulatory hormone glucagon, to suppress the insulin-sensitizing hormone adiponectin, which blocks the hepatic signal for insulin at the level of phosphoinositide 3-kinase and inhibits insulin secretion [9]. Ghrelin increases the secretion of glucagon in the endocrine pancreas in vitro, but whether this occurs in vivo is still unknown [45].

Studies have shown that inactivation of the pre-proghrelin gene in lean rats reduces the level of fasting glycemia and endogenous glucose production, and increases the level of glucose-stimulated insulin compared to wild-type rats. These data indicate that ghrelin limits gluconeogenesis and the synthesis of glycogen mediated by insulin [45]. In addition, the suppression of ghrelin in *ob*/*ob* rat models of diabetes reduces glycemia and fasting insulin and improves glucose tolerance [43, 45, 46].

In humans, the plasma concentration of ghrelin is inversely correlated with the degree of adiposity and changes in BMI and body weight. Obese individuals have a lower circulating level of ghrelin, though this level is increased if these individuals undergo diet-induced weight loss [9, 46]. The effect of bariatric surgery on the plasma concentration of ghrelin is controversial. An increase in ghrelin is expected with weight loss, but this increase does not always occur, and reduction in plasma ghrelin levels is also observed [17, 45]. These apparently paradoxical findings may be explained by the application of different surgical techniques.

The highest ghrelin concentration occurs in the gastric fundus. Its production decreases when this area is disconnected, as may occur after bariatric surgery involving proximal gastric resection. Plasma ghrelin is also significantly reduced after RYGB, although it increases in obese individuals who experience similar levels of diet-induced weight loss. Permanent absence of food in the stomach and duodenum in the context of gastric bypass may result in a continuous stimulatory signal to inhibit ghrelin, favoring weight loss after surgery [47]. On the other hand, the plasma concentration of ghrelin is high after procedures that leave the gastric fundus and the vagal nerve intact, such as after adjustable gastric band implantation. However, if a small amount of ghrelin-producing tissue remains after surgery, postoperative plasma concentration of ghrelin may not change [17, 48]. Considering the physiological properties of ghrelin, a decrease in plasma levels after surgery likely plays an important role in the mediation of weight loss and the beneficial metabolic effects of bariatric surgery [9].

The recent Swiss multicenter randomized study [49] compared, in 217 obese patients, two surgical techniques: RYGB (n:110), a mixed technique, and laparoscopic sleeve gastrectomy ((LSG) n:107) a restrictive technique. There were no differences in body mass index (BMI), age, comorbidities and feeding behavior between the groups. The mean operative time was shorter for LSG than for RYGB. Complications (<30 days) had a tendency to occur more often in RYGB than LSG (p=0.067). However, the difference of serious complications was not statistically significant (p=0.21). Body weight loss was similar in both groups after one year of surgery (p=0.2). The authors concluded that LSG could be performed in shorter time than RYGB and have a trend toward fewer complications than RYGB. Both techniques showed similar efficiency in body weight loss and improvement in associated comorbidities, with the exception for gastroesophageal reflux, better resolved after RYGB. It calls attention that T2D was efficiently treated by both surgeries techniques without differences among them [49].

SG improves glucose metabolism as effectively as RYGB. The underlying mechanism is still not clear but, Zhang W. et al, [50] hypothesized that the ghrelin hormone could be involved in this mechanism. Inactive ghrelin (des acyl ghrelin) is activated to acyl ghrelin in the stomach through the action of the ghrelin *O*-acyltransferase enzyme (GOAT) - the active form of ghrelin. The inhibition of GOAT activity may be consequence of SG technique. Moreover, ghrelin may exerts its orexigenic action through specific modulation of Sirtuin1 (SIRT1)/p53 and AMP-actived protein kinase (AMPK) pathways, which increase the agouti-related protein (AgRP) and neuropeptide Y (NPY) expression in the hypothalamic arcuate nucleus (ARC). Ghrelin triggers a sharp rise at the hypothalamic regulation of mammalian target of rapamycin signaling pathway (mTOR) [51]. There is reciprocal interaction between mTOR pathways and ghrelin/GOAT axis. After SG, the reduced food intake is signaled as peripheral negative energy balance, with the consequent mTOR activity inhibition. This would have stimulated the gastric ghrelin production and secretion and food intake signaling in the hypothalamus. However, the stomach cannot handle this situation, as the ghrelin-producing X/A cell volume is significantly reduced in SG.

Martins L, et al, [51] demonstrated that the central inhibition of mTOR signaling with rapamycin decreased the orexigenic action of ghrelin and normalized mRNA expression of NPY and AgRP as well as its major downstream transcription factors, ie, the protein cAMP response-element binding protein (pCREB) and forkhead box O1 (FoxO1 total and phosphorylated). In summary, mTOR activation is a hypothalamic, mainly ARC-located, mechanism mediating ghrelin’s action on feeding through increased AgRP and NPY gene expression. The activation of hypothalamic mTOR signaling seems to be a mediator of food intake, of potential importance for the understanding and treatment of obesity. Therefore, the GOAT activity down regulation induces activation of mTOR. Secretion of insulin induced by glucose can be enhanced and resolution of T2D achieved [50, 51].

### Cholecystokinin

Cholecystokinin (CCK) acts in satiety control, vesicular contraction, pancreatic and gastric acid secretion, and glucose homeostasis. The CCK peptide is produced by the I cells present mainly in the duodenum and jejunum and is secreted in response to nutrients in the intestinal lumen, particularly lipids and proteins [43, 52]. CCK levels increase rapidly and peak 15 min after meals [53]. CCK has two main receptors, CCK1R and CCK2R. CCK1R appears to be responsible for reduction of food intake [42], while CCK2R mediates the control of glucose homeostasis by the pancreas. *In vitro*, CCK stimulates glucagon release by human pancreatic islets and increases pancreatic β cell proliferation [43]. However, experimentally, CCK also stimulates insulin secretion in a glucose-dependent manner. In addition, the infusion of a form of CCK with eight amino acids (CKK-8) in T2D subjects increases the plasma concentration of insulin and reduces postprandial glucose after meals [43]. Few studies in humans have linked CCK with bariatric surgery. A pioneering study that evaluated patients receiving RYGB failed to detect changes in the plasma concentration of CCK 10 months after the surgery [53].

Few studies were conducted to evaluate CCK. However, Mumphrey et al, [37] in an experimental study assessed the long term effect of RYGB in enteroendocrine cells. Numbers of CCK cells were significantly increased in the Roux and common limbs, but not the biliopancreatic limb in RYGB rats. The findings suggest that the number of enteroendocrine cells increases passively as the gut adapts, and that the increased total number of I cells is likely to contribute to the higher circulating levels of CCK, potentially leading to suppression of food intake and stimulation of insulin secretion [37].

Until recently, CCK has not received much attention, probably because no changes in the postprandial CCK-response to a mixed protein-fat meal were reported 6 months after RYGB in an early study [38]. However, recently it was reported that postprandial CCK response was significantly increased 2 weeks after RYGB [39]. Difference in the postprandial CCK response was also found between patients with RYGB or SG. RYGB had a significant duodenal exclusion effect on CCK [54]. One year postoperatively, CCK concentrations after test meals increased less in the RYGB group than SG group, with the latter show significantly higher maximal CCK concentrations [40]. More consistent findings were reported in patients after jejuno-ileal bypass surgery, with postprandial CCK levels significantly increased 3 months [55] and even 20 years after surgery [56]. The proximal jejunum was anastomosed to the terminal ileum. This technique has been confirmed as effective for weight loss, though serious complications led to abandoning it in the 1970s [57].

### Incretins

Incretins are enteric hormones considered to be insulinotropic due to their capacity to stimulate postprandial insulin secretion. The incretin concept is based on the observation of a greater insulin response to oral glucose compared to intravenous glucose. Substances derived from the intestine and released as a consequence of oral nutrient intake (incretins) were considered potential secretagogues of insulin [58]. These findings support the hypothesis that the gluco-regulatory process results from the interaction of pancreatic hormones (insulin and glucagon) and intestinal hormones; in addition, these findings reinforce the concept that T2D may arise from different hormone systems [59].

The two main incretins are gastric inhibitory polypeptide (GIP) and glucagon-like peptide-1 (GLP-1) [58, 60]. These proteins act directly on pancreatic β cells, which express G protein-coupled receptors (GPCRs) for both GLP-1 and GIP. Stimulation of these receptors increases cyclic adenosine monophosphate (cAMP) concentrations, which, in turn, increases insulin secretion under permissible conditions, such as the presence of high plasma glycemia [61].

GIP is a 42-amino acid peptide cleaved from its precursor peptide, proGIP. Its nomenclature arose from its ability to inhibit gastric acid secretion. Both active [GIP (1–42)] and inactive [GIP (3–42)] forms of GIP are produced by K cells in the duodenum and jejunum under the presence of glucose and fat in the duodenum. GIP also stimulates insulin secretion and is currently being called glucose-dependent insulinotropic polypeptide [58, 60]. No effects on glucagon secretion have been experimentally reported during episodes of hyperglycemia, suggesting that GIP-mediated glucagon secretion depends on hypoglycemia [62].

**Figure 1:**
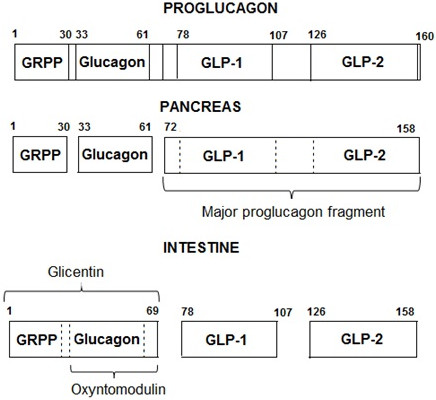
**Post-translational processing of proglucagon in different tissues.** Legend: Abbreviations - GRPP: glicentin-related pancreatic peptide; GLP-1: glucagon-like peptide 1; GLP-2: glucagon-like peptide 2. Adapted from [63].

GLP-1 and GLP-2 are cleaved from the precursor proglucagon by intestinal endocrine L cells, which are located mainly in the distal ileum and colon [60]. Proglucagon is mainly expressed in the L cells of the intestine and α cells of the endocrine pancreas. The primary transcripts and translation products of the *proglucagon* gene are identical in the two types of cells, but post-translational processing of proglucagon differs in a tissue-specific manner, resulting mainly in a bioactive glucagon peptide in the pancreas and GLPs in the intestine (Figure 1) [60, 63].

In the enteroendocrine L cells, GLP-1 and GLP-2 are produced by prohormone convertase 1/3 (PC 1/3) action. Post-translational processing leads to multiple circulating forms of GLP-1: the inactive forms GLP-1 (1–37) and GLP-1 (1–36) and the biologically active forms, including the N-terminal peptides GLP-1 (7–37) and GLP-1 (7–36) amide. Both active peptides are powerful insulinotropics. On the other hand, GLP-2 has powerful intestinotropic properties on the proliferation and apoptosis of the intestinal mucosa, but does not stimulate insulin secretion [44, 60, 62, 64, 65].

GLP-1 (7–37) largely stimulates pancreatic β cells to secrete insulin after postprandial glycemic increases [42, 44]. GLP-1 (7–36) is the most frequent active form of GLP-1 in the circulation and reduces serum levels of glucose by stimulating insulin secretion [66]. GLP-1 preserved and even increased β cell mass in cultures of isolated human pancreatic islets, while the destruction of GLP-1 receptors increased β cell apoptosis [46, 62]. The fasting levels of GLP-1 are low, but increase after the ingestion of mixed meals or meals rich in fat and carbohydrates. In addition to stimulating insulin secretion, GLP-1 suppresses the release of glucagon, decelerates gastric emptying, improves insulin sensitivity, and reduces food consumption [58, 60].

In humans, GIP and GLP-1 concentrations in the circulation increase within 15 min after a meal and reach a peak (~200 to 50 pmol/L, respectively) between 30 to 45 min after a meal, returning to baseline concentrations after 2–3 hours. The interaction between the incretins GLP-1 and GIP is responsible for approximately 50% of postprandial insulin increase. Both incretins have a half-life of 3 to 5 min due to the enzymatic actions of dipeptidyl peptidase IV (DPP-IV), which quickly converts the active forms of GLP-1 and GIP into their inactive metabolites [64, 65].

DPP-IV, also known as CD26, is a ubiquitous enzyme widely expressed in several tissues and cell types, including the kidneys, lungs, adrenal gland, liver, intestines, testes, pancreas, central nervous system, and on the surface of lymphocytes and macrophages. In addition to the form linked on the surface of cells, a soluble protein form of this enzyme is found in the circulation [60, 67]. Because of the wide distribution of DPP-IV, GLP-1 undergoes rapid degradation. Approximately 25% of the secreted hormone reaches the venous circulation in an intact form, 40% to 50% is cleaved in the liver, and only 10% to 15% reaches the peripheral circulation as intact GLP-1 [62].

Patients with T2D are incretin-deficient. This deficit appears to occur due to the reduced secretion of GLP-1 and to the impaired insulinotropic effect of GIP. In these patients, the plasma concentrations of GLP-1 are diminished, but the biological effect of GLP-1 in stimulating insulin secretion is preserved. Explanations for these observations include defective expression and downregulation of GIP receptors in pancreatic β cells, while the underlying mechanism of diminished GLP-1 secretion is not yet known [48]. There is a lack of substantial studies evaluating the effect of bariatric surgery on GLP-1 levels, but available results suggest that postprandial GLP-1 consistently increases soon after malabsorptive bariatric surgery, while purely restrictive procedures do not change postprandial GLP-1 levels [46].

In opposition to GLP-1, the plasma concentrations of GIP are normal in T2D patients, but the effect of GIP on insulin secretion is affected [58, 68]. Some studies report the effect of bariatric surgery on GIP plasma levels. Under fasting conditions, this hormone has been found to be either reduced or unchanged after surgical interventions that lead to the malabsorption of food. Postprandial levels of GIP are reduced in obese patients 2 weeks after jejunoileal bypass [69] or RYGB. Postprandial levels of GIP are also reduced in obese patients with T2D after biliopancreatic derivation [70, 71], but increased 1 month after gastric bypass [72, 73]. Studies evaluating the effect of restrictive surgical procedures on plasma levels of GIP reported no changes at least 23 months after implantation of an adjustable gastric band [74, 75]. Recently it has been speculated that GIP stimulates pancreatic glucagon secretion and stimulates pancreatic β cell growth, leading a glucagonotropic effect in T2D patients [34, 35].

### Enteroglucagon

The enteroglucagon peptide, also coded by the proglucagon gene, is expressed primarily in the L cells of the distal intestine. The term enteroglucagon refers to the intestinal GLPs—mainly glicentin and oxyntomodulin (OXM) [76].

Glicentin is a 69-amino acid peptide with no clearly defined biological activity. This hormone may stimulate the secretion of insulin and inhibit glucagon, but glicentin also appears to inhibit the secretion of gastric acid and to regulate intestinal motility [76, 77]. However, with the discovery of the structure of proglucagon, some researchers consider glicentin only a discarded metabolite of proglucagon after the cleavage of GLP-1 and GLP-2 [76, 78].

OXM is a 37-amino acid peptide that mainly reduces gastric acid secretion, but it also acts directly in the hypothalamic centers to reduce appetite and caloric ingestion. OXM increases insulin secretion and prevents pancreatic β cell apoptosis. Similar to GLP-1, OXM is inactivated rapidly by DPP-IV [53]. OXM can decrease serum concentrations of ghrelin by approximately 15% to 20% in rodents and by 44% in humans [21]. The specific OXM receptor has not yet been identified, although it acts as a double agonist for the GLP-1 receptor with a very low affinity (50 times weaker than that of GLP-1) [52, 76, 79, 80].

A randomized study evaluated OXM levels in 20 obese women with T2D 1 month after RYGB (*n* = 10) and diet-induced equivalent weight loss (*n* = 10). The oral glucose tolerance test (OGTT) was accompanied by an increase of OXM only after bariatric surgery. The increase of OXM was significantly correlated with an increase in hormones GLP-1 and PYY (3–36). This result is not surprising, as long as OXM is secreted along with these hormones by the intestinal L cells. The authors concluded that changes in plasma concentrations of OXM occurred mainly in response to the surgical intervention and not as a consequence of weight loss, which may partly explain the success of the surgery in regards to T2D resolution [79].

Further studies about the effect of different bariatric surgery techniques on enteroglucagon expression in the small intestine are necessary.

### Peptide YY

Peptide tyrosine tyrosine (PYY) is composed of 36 amino acids, with tyrosine (Y) as the first and last in the sequence [21]. This hormone belongs to the pancreatic polypeptide (PP) family and is released by the intestinal L cells, mainly in the ileum, colon, and rectum [44, 81]. The action of PYY is to inhibit gastrointestinal motility and exocrine pancreas and gastric secretions [81]. The circulating concentration of PYY is low during fasting, increases rapidly after a meal, with a peak after 1 to 2 hours, and remains high for several postprandial hours [59]. PYY secretion is proportional to the caloric density of the food eaten, and higher levels are observed after consuming lipids and carbohydrates [17].

After the secretion of PYY, DPP-IV cleaves the N-terminal of its orexigenic form (1–36), producing its anorectic form (3–36) [78]. The activity of DPP-IV is increased in obese individuals. High plasma concentrations of this enzyme, with resulting increases in PYY (3–36) [82], are observed after bariatric surgery. In fact, a postprandial response of increased PYY is evident in the earliest post-surgical period, specifically after malabsorptive bariatric surgery, even before weight loss occurs. Le Roux et al. [83] and Morínigo et al. [84] observed increased postprandial levels of PYY and GLP-1 at 2 days and within 6 months after RYGB, respectively.

The truncate form PYY (3–36), which is released after a meal, induces satiety via the Y2 receptor expressed in the hypothalamus [45]. The main peripheral action of PYY is to reduce lipolysis, but PYY also increases insulin sensitivity by decreasing the concentration of circulating fatty acids. Studies in rats have shown that PYY (3–36) reinforces the action of insulin in improving glycemic control, regardless of eating habits and weight loss [85, 86].

**Figure 2:**
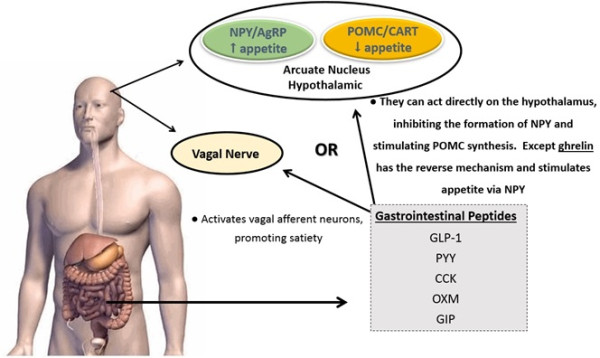
**Gastrointestinal hormones and hypothalamic signaling.** Legend: Abbreviations – NPY: Neuropeptide Y; AgRP: agouti-related protein; POMC: Proopiomelanocortin; CART: cocaine and amphetamine-regulated transcript; GLP-1: Glucagon Like Peptide-1; PYY: Peptide YY; CCK: Cholecystokinin; OXM: Oxyntomodulin; GIP: Glucose-depedent insulinotropic polypeptide.

Regarding the mechanisms of gastrointestinal hormonal action in the hypothalamic signaling, the beginning of the food intake process results in the release of anorexigenic hormones, such as: GLP-1, PYY, CCK and GIP. This result in the activation of neuropeptides, such as neurons Proopiomelanocortin (POMC) and cocaine and amphetamine-regulated transcript (CART) that occurs in the postprandial state, decreasing the appetite. On the other hand, the release of the orexigenic hormone ghrelin and the activation of the neuropeptides NPY and AgRP occurs in the fasting state, increasing the appetite. The gastrointestinal peptides can activate vagal afferent neurons or can act directly on the hypothalamus [42, 87] (Figure 2).

### Bariatric surgery: remission of T2D and its recurrence

According to Clinical Practice Guidelines for the Perioperative Nutritional, Metabolic, and Nonsurgical Support of the Bariatric Surgery Patient—2013 [88], significant body weight regain or failure to lose body weight should prompt evaluation or (a) decreased patient adherence with lifestyle modification, (b) evaluation of medications associated with body weight gain or impairment of body weight loss, (c) development of maladaptive eating behaviors, (d) psychological complications, and (e) radiographic or endoscopic assessment to assess pouch enlargement, anastomotic dilation, formation of a gastric fistula among patients who underwent a RYGB, or inadequate band restriction among patients who underwent AGB. In patients with or without complete resolution of T2D, continued management should be performed. Routine metabolic and nutritional monitoring is recommended after all bariatric surgical procedures [88].

A meta-analyse has found the resolution of T2D in approximately 80% of obese diabetic patients who have had RYGB and BPD–DS surgeries. Conversely, the resolution rates are slightly lower with restrictive-only procedures but are still greater than any available diabetic medical therapy [89]. A recent randomized study compared, for T2D 150 patients, the effect of only intensive medical therapy versus intensive medical therapy plus RYGB or SG surgery. The authors assessed outcome after 3 years post randomization. The primary end point was a glycated hemoglobin level of 6.0% or less, which was met by 5% of the patients in the medical-therapy group, as compared with 38% of those in the RYGB (p<0.001) and 24% of those in the SG group (p=0.01). The use of medications for glycemic control, was lower in the surgical groups than in the medical-therapy group [90].

The beneficial effects on glucose homeostasis after bariatric surgery are likely due, in large part, to the effect of body weight loss on insulin sensitivity and possibly additional beneficial effects on β cell function and the metabolic response to feeding. Furthermore, it has been observed that those obese patients with shorter duration of T2D (less than 5 years), mild forms of T2D (no insulin requirements) and having the greatest body weight loss are more likely to have a remission of T2D when compared with those with longer duration of the disease or chronic T2D (more than 10 years), and are on insulin therapy [91]. The remission rate in patients with T2D for ≤5 years was 95% compared to 75% in patients who had diabetes for 6 to 10 years and 54% in those who had diabetes for more than 10 years (p<0.001) [92, 93]. It is accepted that the duration and severity of T2D is inversely correlated to the likelihood to its remission after surgery, may be due, in part, by chronic and acquired β cell dysfunction [91].

Hall et al. [28] showed that patients with HbA1c >10% had a 50% rate of remission compared to 77.3% with an HbA1c of 6.5–7.9%. The mean duration of T2D preoperatively was 5.5±7 years. A preoperative duration of T2D >10 years was shown to significantly reduce the chances of remission of T2D. A short period of time with diabetes and good glycemic control before surgery may result in a better remission rate for diabetes, suggesting that bariatric surgery should be performed earlier in T2D patients than it is done nowadays. In addition, not all bariatric procedures have the same effect on body weight and glycemic homeostasis for T2D control [28].

Chikunguwo et al. [92] in a retrospective long-term study analyzed 177 T2D patients who had undergone RYGB. Early remission of T2D occurred in 89% of patients after RYGB and T2D recurrence happened in 43.1%. Durable (>5-year) resolution of T2D was greatest in the patients who originally had either controlled their T2D with diet (76%) or oral hypoglycemic agents (66%). The rate of T2D remission was more likely to be durable in men (p=.00381). Body weight regain was statistically significant, but a weak predictor, of T2D recurrence [92].

DiGiorgi studying 42 RYGB patients with T2D and > 3 years of follow-up has shown that T2D had either resolved or improved in all patients (64% and 36%, respectively); but in 24% recurred or worsened. The patients with recurrence or worsening had had a lower preoperative BMI than those without recurrence or worsening (p=.05), regained a greater percentage of their body lost weight (p =.002), had a greater body weight loss failure rate (p=.03), and had greater postoperative glucose levels (p=.0002). Patients who required insulin or oral medication before RYGB were more likely to experience improvement rather than resolution [93].

A retrospective study comparing the long-term effects of SG, RYGB and AGB on T2D, was performed with 60 morbidly obese T2D patients. The mean follow-up period was 36 months. The T2D resolution rate was 60.8% for the AGB technique, 81.2% for RYGB and 80.9% for the SG technique. The resolution rate remained constant at 36-month follow-up evaluation in both the RYGB and SG group, but not at AGB group. The authors have concluded that, for 3-year follow-up, all three bariatric procedures are effective in treating diabetes, but the AGB procedure was the least effective. The antidiabetic effect was similarly earlier after RYGB and SG compared with AGB. This difference may indicate that a hormonal mechanism may be involved, independent of body weight loss [94].

**Figure 3:**
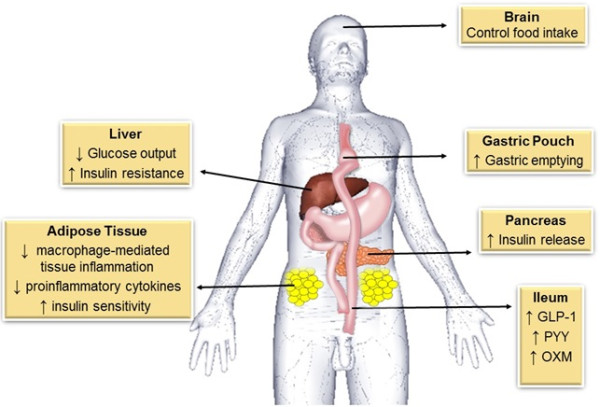
**General schematic representation of the mechanisms after RYGB that contribute for glycemic homeostasis and type 2 diabetes control.** Legend: RYGB can control food intake through gastrointestinal hormones action. The reduced gastric pouch favors gastric emptying. In the liver, there is insulin sensitivity improvement, with decrease of insulin resistance and glucose output. In adipose tissue occurs decrease of inflammation and production oh proinflammatory cytokines, improving insulin sensitivity. In the pancreas the release of insulin increase and all these change leads to T2D remission. Abbreviations: GLP-1: Glucagon Like Peptide-1; PYY: Peptide YY; OXM: Oxyntomodulin.

Considered the gold standard, RYGB is the most commonly used bariatric surgery technique worldwide (Figure 3).

### Critical points of RYGB

A recent systematic review and meta-analysis for bariatric surgery confirmed the substantial positives effects in obesity and metabolic conditions, however, calls attention for the risks of complication, reoperation, and death. A total of 164 studies were included (37 randomized clinical trials and 127 observational studies). Analyses included 161.756 patients. The mortality rate within 30 days was 0.08%, and after 30 days was 0.31%. The complication rate was 17%. The reoperation rate was 7%. RYGB was more effective in body weight loss however was associated with more complications. AGB had lower mortality and complication rates; yet, the reoperation rate was higher than RYGB. SG appeared to be more effective in weight loss than AGB and comparable with RYGB. In general, surgical complications included all adverse events associated with bariatric surgery, such as bleeding, stomal stenosis, leak, vomiting, reflux, gastrointestinal symptoms, and nutritional and electrolyte abnormalities [95].

### Final considerations

There are several hypotheses regarding metabolic changes induced by bariatric surgery, which may be associated with T2D remission. All of the hypotheses involve or can be associated with the metabolic properties of the intestine. Two hypotheses consistently suggest that the pathophysiology of T2D involves an imbalance in incretins and the release of other insulinotropic hormones (considered anti-diabetogenic signals; hindgut hypothesis), as well as the release of still-undetermined anti-incretin factors (considered diabetogenic signals; foregut intestine hypothesis) [34].

Glucagon is considered a strong candidate for a diabetogenic signal because T2D is associated with little secretion of insulin and excessive release of glucagon [78]. Glucagon is originated by the cleavage of proglucagon expressed by both α pancreatic cells and L intestinal cells. Therefore, glucagon can be produced by the intestine, mainly by the distal portion of the small intestine. As illustrated in Figure 1, the transcription and translation of the proglucagon gene vary in the pancreas and intestine. In the pancreas, these processes result in the production of the hormones glucagon, glicentin-related pancreatic peptide (GRPP), and major proglucagon fragment (MPGF). In the intestine, this process results in the production of the hormones GLP-1, GLP-2, glicentin, and OXM [33, 34, 36].

The GIP hormone is one important factor that may contribute to the increase of glucagon in T2D patients. In these patients, GIP has less insulinotropic and more glucagonotropic actions and was found to be decreased after malabsorptive bariatric surgery. The reduced glucagonotropic signaling of GIP could contribute to the restoration of normal glucose tolerance observed after this kind of procedure. Thus, GIP has been considered as a diabetogenic signal produced by the duodenum that is excluded after surgery [29, 33–36].

Data of recurrence of T2D after RYGB and others techniques are still limited. However the weight is observed in approximately 50% of the patients (46% within 24 months and 63.6% within 48 months) [96]. Chikunguwo SM et al, showed that weigh regain occurred between 18 and 24 montgs after surgert in 30% of patients [92]. Some factors can influence in weigh regain after surgery, such as, type of surgery performed, in which we realized in clinical practice that the more malabsorptive technique, lower the regained weight; presence of eating disorders; patient adherence to the support groups and pre surgical BMI [96]. Although many studies discuss only remission rates of T2D after bariatric surgery, dates about durability of this remission are limited. Some studies show that the durability of T2D remission is correlated with sustained weigh loss. This remission of T2D is more durable in patients with less severe diabetes in preoperative period, using either diet or oral hypoglycemic agents to glycemic control. Patients with use of insulin have more chance of T2D recurrence after bariatric surgery. Durable remission correlated most closely with an early disease stage of T2D [92].

## Conclusion

Obesity is associated with an increased risk of developing insulin resistance and T2D. The systemic insulin resistance in obesity can be initiated largely in adipose tissue, and macrophage-mediated tissue inflammation is a core mechanism of dysfunction in adipose tissue. Adipose tissue can communicate with the liver and other organs, such as, muscle and pancreas, and release proinflammatory cytokines leading to insulin sensitivity. When insulin resistance is accompanied by dysfunction of pancreatic islet β-cells, failure to control blood glucose levels results. Abnormalities in β-cell function are therefore critical in defining the risk and development of T2D.

Bariatric surgery involves changes in gastrointestinal anatomy and has been shown to be effective in the treatment of obesity and obesity-associated comorbidities. The early remission of T2D after RYGB appears to be a direct consequence of gastrointestinal anatomy rebuilding and not exclusively a result of decreased food intake and/or weight loss. Until now, is not clear which hypothesis is more accept and consistent for improvement in glycemic control after surgery. However, the hindgut hypothesis has been suggested as a potent mechanism to TDM remission, because the plasma levels of hormones GLP-1 and PYY are increased in obese patients with T2DM after surgeries with a malabsorptive component, such as RYGB surgeries, as discussed previously by our group in a recent review. We believe that in addition to PYY and GLP-1 are strong candidates for this hypothesis, another unknown factors release by lower intestine, can be involved in glycemic normalization following RYGB.

Current evidence, although restricted to a small number of studies, consistently supports a potential role for some intestinal hormones in improving diabetes after RYGB, highlighting the importance of understanding the intestine as a metabolically active organ that may be managed in the future to improve health.

## Abbreviations

T2D: Type 2 diabetes mellitus
BMI: Body mass index
CCK: Cholecystokinin
GHS-R: Growth hormone secretagogue-receptor
GIP: Gastric inhibitory polypeptide
GLP-1: Glucagon-like peptide-1
GLP-2: Glucagon-like peptide-2
GPCRs: G protein-coupled receptors
cAMP: Cyclic adenosine monophosphate
DPPIV: Dipeptidyl peptidase IV
OXM: Oxyntomodulin
OGTT: Oral glucose tolerance test
PYY: Peptide tyrosine tyrosine
PP: Pancreatic polypeptide
MPGF: Major proglucagon fragment
GRPP: Glicentin-related pancreatic peptide
RYGB: Roux-en-Y gastric bypass
AGB: Adjustable gastric band
SG: Sleeve gastrectomy
LSG: laparoscopic sleeve gastrectomy
DJB: Duodenojejunal bypass
BPD: Biliopancreatic diversion
BPD-DS: Biliopancreatic diversion with duodenal switch
PP: postprandial
GOAT: *O*-acyltransferase enzyme
SIRT1: Sirtuin1
AMPK: AMP-actived protein kinase
AgRP: agouti-related protein
NPY: neuropeptide Y
ARC: arcuate nucleus
mTOR: mammalian target of rapamycin
pCREB: response-element binding protein
FoxO1: forkhead box O1
POMC: Proopiomelanocortin
CART: cocaine and amphetamine-regulated transcript.

## References

[1] Lima-Costa MF, Peixoto SV, Firmo JO, Uchoa E (2007). Validity of self-reported diabetes and its determinants: evidences from the Bambui study. Rev Saude Publica.

[2] Lapolla A, Dalfra MG, Fedele D (2008). Pregnancy complicated by type 2 diabetes: an emerging problem. Diabetes Res Clin Pract.

[3] Holst JJ (2013). Enteroendocrine secretion of gut hormones in diabetes, obesity and after bariatric surgery. Curr Opin Pharmacol.

[4] Flatt PR (2007). Effective surgical treatment of obesity may be mediated by ablation of the lipogenic gut hormone Gastric Inhibitory Polypeptide (GIP): evidence and clinical opportunity for development of new obesity-diabetes drugs?. Diab Vasc Dis Res.

[5] Bandeira F, Macedo G, Caldas G, Griz L, Faria MS (2003). Endocrinologia e Diabetes Mellitus.

[6] Osborn O, Olefsky JM (2012). The cellular and signaling networks linking the immune system and metabolism in disease. Nat Med.

[7] Mingrone G, Castagneto-Gissey L (2009). Mechanisms of early improvement/ resolution of type 2 diabetes after bariatric surgery. Diabetes Metab.

[8] Li B, Zhou X, Wo J, Zhou H (2013). From gut changes to type 2 diabetes remission after gastric bypass surgeries. Front Med.

[9] Karra E, Yousseif A, Batterham RL (2010). Mechanisms facilitating weight loss and resolution of type 2 diabetes following bariatric surgery. Trends Endocrinol Metab.

[10] Wilson JB, BaWJP MD (2010). Durable remission of diabetes after bariatric surgery: What is the underlying pathway?. Insulin.

[11] American Diabetes Association (2009). Standards of medical care in diabetes—2009. Diabetes Care.

[12] ASMBS: American Society for Metabolic and Bariatric Surgery (2014). Bariatric Surgery Procedures.

[13] Papamargaritis D, Le Roux CW, Sioka E, Koukoulis G, Tzovaras G, Zacharoulis D (2013). Changes in hormone profile and glucose homeostasis after laparoscopic sleeve gastrectomy. Surg Obes Relat Dis.

[14] Rubino F, Marescaux J (2004). Effect of duodenal–jejunal exclusion in a non-obese animal model of type 2 diabetes: a new perspective for an old disease. Ann Surg.

[15] Paik KY, Kim W, Song KH, Kwon HS, Kim MK, Kim E (2012). The preliminary clinical experience with laparoscopic duodenojejunal by pass for treatment of type 2 diabetes mellitus in non-morbidly obese patients: the 1-year result in a single institute. Surg Endosc.

[16] Tsoli M, Chronaiou A, Kehagias I, Kalfarentzos F, Alexandrides TK (2013). Hormone changes and diabetes resolution after biliopancreatic diversion and laparoscopic sleeve gastrectomy: a comparative prospective study. Surg Obes Relat Dis.

[17] Thomas S, Schauer P (2010). Bariatric surgery and the gut hormone response. Nutr Clin Pract.

[18] Scopinaro N, Gianetta E, Adami GF, Friedman D, Traverso E, Marinari GM, Cuneo S, Vitale B, Ballari F, Colombini M, Baschieri G, Bachi V (1996). Biliopancreatic diversion for obesity at eighteen years. Surgery.

[19] Hess DS, Hess DW (1998). Biliopancreatic diversion with a duodenal switch. Obes Surg.

[20] Wilson JB, Pories WJ (2010). Durable remission of diabetes after bariatric surgery: What is the underlying pathway?. Insulin.

[21] Rubino F, Schauer PR, Kaplan LM, Cummings DE (2010). Metabolic surgery to treat type 2 diabetes: clinical outcomes and mechanisms of action. Annu Rev Med.

[22] Cummings DE (2009). Endocrine mechanisms mediating remission of diabetes after gastric bypass surgery. Int J Obes (Lond).

[23] Dixon JB, O’Brien PE, Playfair J, Chapman L, Schachter LM, Skinner S, Proietto J, Bailey M, Anderson M (2008). Adjustable gastric banding and conventional therapy for type 2 diabetes: a randomized controlled trial. JAMA.

[24] Kashyap SR, Gatmaitan P, Brethauer S, Schauer P (2010). Bariatric surgery for type 2 diabetes: weighing the impact for obese patients. Cleve Clin J Med.

[25] Buchwald H, Avidor Y, Braunwald E, Jensen MD, Pories W, Fahrbach K, Schoelles K (2004). Bariatric surgery: a systematic review and meta-analysis. JAMA.

[26] Rubino F (2008). Is type 2 diabetes an operable intestinal disease? A provocative yet reasonable hypothesis. Diabetes Care.

[27] Scopinaro N, Marinari GM, Camerini GB, Papadia FS, Adami GF (2005). Specific effects of biliopancreatic diversion on the major components of metabolic syndrome: a long-term follow-up study. Diabetes Care.

[28] Hall TC, Pellen MG, Sedman PC, Jain PK (2010). Preoperative factors predicting remission of type 2 diabetes mellitus after Roux-en-Y gastric bypass surgery for obesity. Obes Surg.

[29] Sala PC, Torrinhas RS, Heymsfield SB, Waitzberg DL (2012). Type 2 diabetes mellitus: a possible surgically reversible intestinal dysfunction. Obes Surg.

[30] Scheen AJ, De Flines J, De Roover A, Paquot N (2009). Bariatric surgery in patients with type 2 diabetes: benefits, risks, indications and perspectives. Diabetes Metab.

[31] Cummings DE, Overduin J, Foster-Schubert KE (2004). Gastric bypass for obesity: mechanisms of weight loss and diabetes resolution. J Clin Endocrinol Metab.

[32] Schauer PR, Burguera B, Ikramuddin S, Cottam D, Gourash W, Hamad G, Eid GM, Mattar S, Ramanathan R, Barinas-Mitchel E, Rao RH, Kuller L, Kelley D (2003). Effect of laparoscopic Roux-en Y gastric bypass on type 2 diabetes mellitus. Ann Surg.

[33] Rubino F, Forgione A, Cummings DE, Vix M, Gnuli D, Mingrone G, Castagneto M, Marescaux J (2006). The mechanism of diabetes control after gastrointestinal bypass surgery reveals a role of the proximal small intestine in the pathophysiology of type 2 diabetes. Ann Surg.

[34] Knop FK (2009). Resolution of type 2 diabetes following gastric bypass surgery: involvement of gut-derived glucagon and glucagonotropic signalling?. Diabetologia.

[35] Nauck MA, Heimesaat MM, Orskov C, Holst JJ, Ebert R, Creutzfeldt W (1993). Preserved incretin activity of glucagon-like peptide 1 [7–36 amide] but not of synthetic human gastric inhibitory polypeptide in patients with type-2 diabetes mellitus. J Clin Invest.

[36] Papamargaritis D, Miras AD, Le Roux CW (2013). Influence of diabetes surgery on gut hormones and incretins. Nutr Hosp.

[37] Mumphrey MB, Patterson LM, Zheng H, Berthoud HR (2013). Roux-en-Y gastric bypass surgery increases number but not density of CCK-, GLP-1-, 5-HT-, and neurotensin expressing enteroendocrine cells in rats. Neurogastroenterol Motil.

[38] Kellum JM, Kuemmerle JF, O’Dorisio TM, Rayford P, Martin D, Engle K, Wolf L, Sugerman HJ (1990). Gastrointestinal hormone responses to meals before and after gastric bypass and vertical banded gastroplasty. Ann Surg.

[39] Jacobsen SH, Olesen SC, Dirksen C, Jørgensen NB, Bojsen-Møller KN, Kielgast U, Worm D, Almdal T, Naver LS, Hvolris LE, Rehfeld JF, Wulff BS, Clausen TR, Hansen DL, Holst JJ, Madsbad S (2012). Changes in gastrointestinal hormone responses, insulin sensitivity, and beta-cell function within 2 weeks after gastric bypass in nondiabetic subjects. Obes Surg.

[40] Peterli R, Steinert RE, Woelnerhanssen B, Peters T, Christoffel-Courtin C, Gass M, Kern B, von Fluee M, Beglinger C (2012). Metabolic and hormonal changes after laparoscopic Roux-en-Y Gastric bypass and sleeve gastrectomy: a randomized, prospective trial. Obes Surg.

[41] Karra E, Batterham RL (2010). The role of gut hormones in the regulation of body weight and energy homeostasis. Mol Cell Endocrinol.

[42] Murphy KG, Bloom SR (2006). Gut hormones and the regulation of energy homeostasis. Nature.

[43] Drucker DJ (2007). The role of gut hormones in glucose homeostasis. J Clin Invest.

[44] Baynes KC, Dhillo WS, Bloom SR (2006). Regulation of food intake by gastrointestinal hormones. Curr Opin Gastroenterol.

[45] Naslund E, Kral JG (2006). Impact of gastric bypass surgery on gut hormones and glucose homeostasis in type 2 diabetes. Diabetes.

[46] Bose M, Olivan B, Teixeira J, Pi-Sunyer FX, Laferrere B (2009). Do Incretins play a role in the remission of type 2 diabetes after gastric bypass surgery: What are the evidence?. Obes Surg.

[47] Cummings DE, Weigle DS, Frayo RS, Breen PA, Ma MK, Dellinger EP, Purnell JQ (2002). Plasma ghrelin levels after diet-induced weight loss or gastric bypass surgery. N Engl J Med.

[48] Vetter ML, Cardillo S, Rickels MR, Iqbal N (2009). Narrative review: effect of bariatric surgery on type 2 diabetes mellitus. Ann Intern Med.

[49] Peterli R, Borbély Y, Kern B, Gass M, Peters T, Thurnheer M, Schultes B, Laederach K, Bueter M, Schiesser M (2013). Early of the Swiss Multicentre Bypass or Sleeve Study (SM-BOSS). A prospective randomized trial comparing laparoscopic sleeve gastrectomy and roux-en-Y gastric bypass. Ann Surg.

[50] Zhang W, Zhu DQ, Qiu M (2012). Hypothesis for resolution of diabetes after sleeve gastrectomy. Med Hypotheses.

[51] Martins L, Fernández-Mallo D, Novelle MG, Vázquez MJ, Tena-Sempere M, Nogueiras R, López M, Diéguez C (2012). Hypothalamic mTOR signaling mediates the orexigenic action of ghrelin. PLoS One.

[52] Halpern ZSC, Rodrigues MDB, Costa RF (2004). Determinantes fisiológicos do controle do peso e apetite. Rev Psiquiatr Clín.

[53] Suzuki K, Simpson KA, Minnion JS, Shillito JC, Bloom SR (2010). The role of gut hormones and the hypothalamus in appetite regulation. Endocr J.

[54] Lee WJ, Chen CY, Chong K, Lee YC, Chen SC, Lee SD (2011). Changes in postprandial gut hormones after metabolic surgery: a comparison of gastric bypass and sleeve gastrectomy. Surg Obes Relat Dis.

[55] Buchan AM, Pederson RA, Koop I, Gourlay RH, Cleator IG (1993). Morphological and functional alterations to a sub-group of regulatory peptides in human pancreas and intestine after jejuno-ileal bypass. Int J Obes Relat Metab Disord.

[56] Naslund E, Gryback P, Hellstrom PM, Jacobsson H, Holst JJ, Theodorsson E, Backman L (1997). Gastrointestinal hormones and gastric emptying 20 years after jejunoileal bypass for massive obesity. Int J Obes Relat Metab Disord.

[57] Griffen WO, Bivins BA, Bell RM (1983). The decline and fall of jejunoileal bypass. Surg Gynecol Obstet.

[58] Chacra AR (2006). Efeito Fisiológico das Incretins.

[59] SBD (2007). Novas perspectivas para o tratamento do diabetes tipo 2: o tratamento do diabetes tipo 2: incretinomiméticos e inibidores da DPP-IV. Rev Bras Med.

[60] Baggio LL, Drucker DJ (2007). Biology of incretins: GLP-1 and GIP. Gastroenterology.

[61] Reimann F (2010). Molecular mechanisms underlying nutrient detection by incretin-secreting cells. Int Dairy J.

[62] Hare KJ (2010). Role of GLP-1 induced glucagon suppression in type 2 diabetes mellitus. Dan Med Bull.

[63] Vilsboll T (2004). On the role of the incretin hormones GIP and GLP-1 in the pathogenesis of Type 2 diabetes mellitus. Dan Med Bull.

[64] Ranganath LR (2008). Incretins: pathophysiological and therapeutic implications of glucose-dependent insulinotropic polypeptide and glucagon-like peptide-1. J Clin Pathol.

[65] Whitson BA, Leslie DB, Kellogg TA, Maddaus MA, Buchwald H, Billington CJ, Ikramuddin S (2007). Entero-endocrine changes after gastric bypass in diabetic and nondiabetic patients: a preliminary study. J Surg Res.

[66] Vahl TP, Paty BW, Fuller BD, Prigeon RL, D’Alessio DA (2003). Effects of GLP-1 (7–36) NH2, GLP (7–37), and GLP-1 (9–36) NH2 on Intravenous Glucose Tolerance and Glucose-Induced Insulin Secretion in Healthy Humans. J Clin Endocrinol Metab.

[67] De Meester I, Korom S, Van Damme J, Scharpe S (1999). CD26, let it cut or cut it down. Immunol Today.

[68] Laferrere B, Heshka S, Wang K, Khan Y, McGinty J, Teixeira J, Hart AB, Olivan B (2007). Incretin levels and effect are markedly enhanced 1 month after Roux-en-Y gastric bypass surgery in obese patients with type 2 diabetes. Diabetes Care.

[69] Jorde R, Burhol PG, Johnson JA (1981). The effect of jejunoileal bypass on postprandial release of plasma gastric inhibitory polypeptide (GIP). Scand J Gastroenterol.

[70] Sirinek KR, O'Dorisio TM, Hill D, McFee AS (1986). Hyperinsulinism, glucose-dependent insulinotropic polypeptide, and the enteroinsular axis in morbidly obese patients before and after gastric bypass. Surgery.

[71] Guidone C, Manco M, Valera-Mora E, Iaconelli A, Gniuli D, Mari A, Nanni G, Castagneto M, Calvani M, Mingrone G (2006). Mechanisms of recovery from type 2 diabetes after malabsorptive bariatric surgery. Diabetes.

[72] Laferrere B, Teixeira J, McGinty J, Tran H, Egger JR, Colarusso A, Kovack B, Bawa B, Koshy N, Lee H, Yapp K, Olivan B (2008). Effect of weight loss by gastric bypass surgery versus hypocaloric diet on glucose and incretin levels in patients with type 2 diabetes. J Clin Endocrinol Metab.

[73] Laferrere B (2009). Effect of gastric bypass surgery on the incretins. Diabetes Metab.

[74] Shak JR, Roper J, Perez-Perez GI, Tseng CH, Francois F, Gamagaris Z, Patterson C, Weinshel E, Fielding GA, Ren C, Blaser MJ (2008). The effect of laparoscopic gastric banding surgery on plasma levels of appetite-control, insulinotropic, and digestive hormones. Obes Surg.

[75] Korner J, Bessler M, Inabnet W, Taveras C, Holst JJ (2007). Exaggerated glucagon-like peptide-1 and blunted glucose-dependent insulinotropic peptide secretion are associated with Roux-en-Y gastric bypass but not adjustable gastric banding. Surg Obes Relat Dis.

[76] Sinclair EM, Drucker DJ (2005). Proglucagon-derived peptides: mechanisms of action and therapeutic potential. Physiology (Bethesda).

[77] Holst JJ (1997). Enteroglucagon. Annu Rev Physiol.

[78] Kahn CR, Weir GC, King GL (2009). Jacobson AM.

[79] Laferrere B, Swerdlow N, Bawa B, Arias S, Bose M, Olivan B, Teixeira J, McGinty J, Rother KI (2010). Rise of oxyntomodulin in respone to oral glucose after gastric bypass surgery in patients with type 2 diabetes. J Clin Endocrinol Metab.

[80] Troke RC, Tan TM, Bloom SR (2014). The future role of gut hormones in the treatment of obesity. Ther Adv Chronic Dis.

[81] Yamada T, Alpers DH, Laine L, Owyang C (1999). Powell DW.

[82] Ballantyne GH, Peptide YY (2006). (1–36) and Peptide YY (3–36): Part II. Changes after Gastrointestinal Surgery and Bariatric Surgery: Part I: Distribution, Release and Action appeared in the last issue. Obes Surg.

[83] Le Roux CW, Welbourn R, Werling M, Osborne A, Kokkinos A, Laurenius A, Lönroth H, Fändriks L, Ghatei MA, Bloom SR, Olbers T (2007). Gut hormones as mediators of appetite and weight loss after Roux-en-Y gastric bypass. Ann Surg.

[84] Morinigo R, Moize V, Musri M, Lacy AM, Navarro S, Marin JL, Delgado S, Casamitjana R, Vidal J (2006). Glucagon-like peptide-1, peptide YY, hunger, and satiety after gastric bypass surgery in morbidly obese subjects. J Clin Endocrinol Metab.

[85] Andreelli F, Amouyal C, Magnan C, Mithieux G (2009). What can bariatric surgery teach us about the pathophysiology of type 2 diabetes?. Diabetes Metab.

[86] van den Hoek AM, Heijboer AC, Corssmit EP, Voshol PJ, Romijn JA, Havekes LM, Pijl H (2004). PYY3-36 reinforces insulin action on glucose disposal in mice fed a high-fat diet. Diabetes.

[87] Pimentel GD, Micheletti TO, Pace F, Rosa JC, Santos RVT, Lira FS (2012). Gut-central nervous system axis is a target for nutritional therapies. Nutr J.

[88] Mechanick JI, Youdim A, Jones DB (2013). Clinical practice guidelines for the perioperative nutritional, metabolic, and nonsurgical support of the bariatric surgery patient—2013 update: cosponsored by American Association of Clinical Endocrinologists, The Obesity Society, and American Society for Metabolic & Bariatric Surgery. AACE/TOS/ASMBS Guidelines. Surg Obes Relat Dis.

[89] Buchwald H, Estok R, Fahrbach K, Banel D, Jensen MD, Pories WJ, Bantle JP, Sledge I (2009). Weight and type 2 diabetes after bariatric surgery: systematic review and meta-analysis. Am J Med.

[90] Schauer PR, Bhatt DL, Kirwan JP, Wolski K, Brethauer SA, Navaneethan SD, Aminian A, Pothier CE, Kim ES, Nissen SE, Kashyap SR, STAMPEDE Investigators (2014). Bariatric surgery versus intensive medical therapy for diabetes — 3-year outcomes. N Engl J Med.

[91] Varela JE (2011). Bariatric surgery: a cure for diabetes?. Curr Opin Clin Nutr Metab Care.

[92] Chikunguwo SM, Wolfe LG, Dodson P, Meador JG, Baugh N, Clore JN, Kellum JM, Maher JW (2010). Analysis of factors associated with durable remission of diabetes after Roux-en-Y gastric bypass. Surg Obes Relat Dis.

[93] DiGiorgi M, Rosen DJ, Choi JJ, Milone L, Schrope B, Olivero-Rivera L, Restuccia N, Yuen S, Fisk M, Inabnet WB, Bessler M (2010). Re-emergence of diabetes after gastric bypass in patients with mid- to long-term follow-up. Surg Obes Relat Dis.

[94] Abbatini F, Rizzelo M, Casella G, Alessandri G, Capoccia D, Leonetti F, Basso N (2010). Long-term effects of laparoscopic sleeve gastrectomy, gastric bypass, and adjustable gastric banding on type 2 diabetes. Surg Endosc.

[95] Chang S-H, Stoll CRT, Song J, Varela JE, Eagon CJ, Colditz GA (2014). The effectiveness and risks of bariatric surgery. An updated systematic review and meta-analysis, 2003–2013. JAMA Surg.

[96] Magro DO, Geloneze B, Delfini R, Pareja BC, Callejas F, Pareja JC (2008). Long-term weight regain aftar gastric bypass: a 5-year prospective study. Obes Surg.

